# Evaluation of the Impacts of Land Use on Water Quality: A Case Study in The Chaohu Lake Basin

**DOI:** 10.1155/2013/329187

**Published:** 2013-07-22

**Authors:** Juan Huang, Jinyan Zhan, Haiming Yan, Feng Wu, Xiangzheng Deng

**Affiliations:** ^1^State Key Laboratory of Water Environment Simulation, School of Environment, Beijing Normal University, Beijing 100875, China; ^2^Institute of Geographic Science and Natural Resource Research, CAS, Beijing 100101, China; ^3^Center for Chinese Agricultural Policy, CAS, Beijing 100101, China

## Abstract

It has been widely accepted that there is a close relationship between the land use type and water quality. There have been some researches on this relationship from the perspective of the spatial configuration of land use in recent years. This study aims to analyze the influence of various land use types on the water quality within the Chaohu Lake Basin based on the water quality monitoring data and RS data from 2000 to 2008, with the small watershed as the basic unit of analysis. The results indicated that there was significant negative correlation between forest land and grassland and the water pollution, and the built-up area had negative impacts on the water quality, while the influence of the cultivated land on the water quality was very complex. Besides, the impacts of the landscape diversity on the indicators of water quality within the watershed were also analyzed, the result of which indicated there was a significant negative relationship between them. The results can provide important scientific reference for the local land use optimization and water pollution control and guidance for the formulation of policies to coordinate the exploitation and protection of the water resource.

## 1. Introduction 

The land use within the watershed has great impacts on the water quality of rivers. The water quality of rivers may degrade due to the changes in the land cover patterns within the watershed as human activities increase [1, 2]. Changes in the land cover and land management practices have been regarded as the key influencing factors behind the alteration of the hydrological system, which lead to the change in runoff as well as the water quality [3, 4].

There have been three waves of the research that tried to reveal the effects of the land use and land cover change on the quality of surface water [5, 6]. The researchers have started to study the linkage between land cover and the river water quality in order to investigate the effects of morphological features of watersheds on the turbidity, dissolved oxygen and temperature of the river water since the early 1960s [7]. The second wave of researches on this topic emerged in the 1970s, focusing on the analysis at the watershed scale [8]. The third wave of these studies have started to take advantage of the remote sensing, GIS, and multivariate analysis to explore the influence of the land cover on the suspended sediment, nutrients and ecological integrity of the stream [9–14].

Related research in China started from the 1980s and mainly focused on the role of the macroscopic characteristics of nonpoint source pollution and urban runoff pollution and the quantitative calculation of pollution loading [15–17]. For example, the research carried out by Guo indicated that it is necessary to take into account both the land use and the land cover pattern simultaneously in the study of the impacts of land use on the water quality [18]. Besides, the export coefficient model and RS and GIS techniques have been applied in the study of the effects of the land use change on the nonpoint source pollution load in the upper reach of Yangtze River [19], and the result indicated that the grassland played a dominant role in influencing the TN and TP in Jinsha River, while cultivated land played a key role in other parts of the study area. There was also research about the relationship between land use types and water quality in Xin' anjiang River based on ArcGIS [20]. The results showed that cultivated land, grassland, and forest land had the most significantly important impacts on TN, TP, and fecal coliform bacteria. On the whole, the previous studies in China have focused on only several lakes such as Tai Lake [21] and Dianchi Lake [22]. Besides, these researches have only taken into account the impacts of the composition of land use types within these basins on the water quality. Only few studies have considered the effects of spatial patterns of land use on the water quality, which could provide evidence on landscape planning and land use management. Therefore, The primary objectives of this paper were (1) to describe the water quality change in the Chaohu Lake Basin from 2000 to 2008; (2) to investigate the land use change in the study area; and (3) to identify the relationship between land use change and water quality.

## 2. Materials and Methods

### 2.1. Study Area

**Figure 1:**
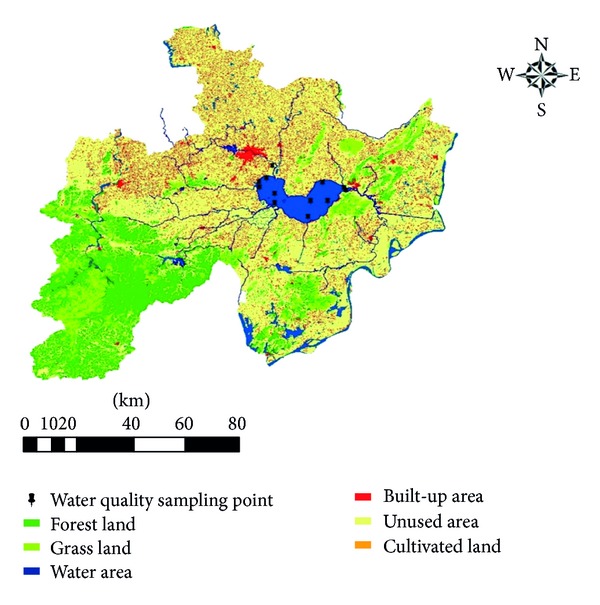
The Chaohu Lake Basin. Water quality points are shown in the figure. Upstream catchment of each water quality sampling point and land use types were delineated.

The Chaohu Lake Basin (Figure 1) is located in the central part of Anhui Province and between 117°16′46′16′′–117°51′54′ 51′′E, 30°43′28′ 43′′–31°25′28′ 25′′N. The Chaohu Lake belongs to the drainage system in the lower reaches of the Yangtze River, and it is the fifth largest freshwater lake in China, with a total watershed area of 13350 km^2^. The total annual inflow from 33 rivers is 4.8 × 10^9^ m^3^ year^−1^ and the total outflow is 3.4 × 10^9^ m^3^ year^−1^. A large portion of the inflow is from Nanfei River, Hangbu River, and Yuxi River. The average annual temperature in the Chaohu Lake Basin ranges between 15°C and 16°C, with a mean annual rainfall of 1100 mm. The Chaohu Lake Basin is one of the most densely populated regions in Anhui Province [23], with a population of more than 9.65 million. The Chaohu Lake Basin also plays an important role in the local economic development, accounting for 24.65% of GDP in Anhui Province. 

### 2.2. Water Quality Situation and Data Resources

The Chaohu Lake Basin was known as the land of fish and rice in the early stage when the local ecological environment was very good and the water quality of Chaohu Lake used to be very fine. However, the hydrological conditions and downstream ecosystems of this lake have been altered since the establishment of Chaohu Dam on Yuxi River [24, 25], with the amount of annual water exchange volume decreasing from 13.6 × 10^8^ m^3^ to 1.6 × 10^8^ m^3^ [26] and the average annual water level falling from 4.3 m to 2.9 m. Besides, with the rapid development of local economy and social activity, the wetlands in the Chaohu Lake Basin were reclaimed or occupied [27]. And water quality of Chaohu Lake has continuously deteriorated due to the large amount of pollution discharge from the local industry, agriculture, and daily life since the late 1970s [28]. Along with that is the outbreak of water bloom, which has aroused great concern of the government. The Chaohu Lake has reached the eutrophic state, with the high concentration of nutrient salts and rapid growth of algae.

The water quality of Chaohu Lake has consciously improved to some degree. In 1997, the Ministry of Environmental Protection of China promulgated the water quality of the five biggest fresh water lakes, among which the situation of Chaohu Lake was the worst. During 1996–1999, the data from water quality monitoring points around Chaohu Lake showed that the percentage of water exceeding the level V had decreased from 80% to 60%. Since 2000, water quality of the main tributaries of Chaohu Lake, including Nanfei River, Shiwuli River, Pai River, and Shuangqiao River, was always exceeding the level V, with ammonia nitrogen as the key pollutant. But the water quality of other tributaries was better, generally fluctuating between level III and level IV (Table 1).

In 2003, Chaohu Lake reached the meso-eutrophication the whole, with the total phosphorus (TP) and total nitrogen (TN) as the main pollutants. The water quality of Chaohu Lake improved slightly in 2005, reaching the meso-eutrophic state on the whole. The mean value of CODmn and TN succeeded in achieving the goal of the tenth Five-year Plan in Chaohu Lake, but the mean value of TP failed. For the moment, Chaohu Lake is known as one of the three most polluted freshwater lakes in China [29]. Since Chaohu Lake serves as the primary drinking water source of Hefei City, it has been ranked as the key lake to be managed.

In this study, the Chaohu Lake Basin was divided into nine watersheds according to the local river systems and the monitoring points were set up over there (Figure 1). There are many variables of water quality available from these monitoring points, including pellucidity, TP, TN, DO, BOD5, and CODcr. But we only selected TP, TN, DO, NH3-N, and CODmn measured in every month from 2000 to 2008. Other water quality variables were also important; we chose these five not because they were more important than others in Chaohu Lake, but because many researches had chosen them, and these water quality variables had the complete data. The average annual values of these variables were also used in view of the seasonal variations of algal species and water quality in Chaohu Lake [30].

### 2.3. Land Use Data

The land use data, which was extracted from the Lantsat TM images (from 2000 to 2008), was provided by the Data Center of the Chinese Academy of Sciences. There are six kinds of land use types, that is, the cultivated land, forest land, grassland, water area, built-up area and unused land. The Landsat ETM images in 2000 and 2005 were interpreted at a scale of 1 : 100,000 and the overall interpretation accuracy of the land use categories reached 92.7% according to the field survey and random sampling check conducted by Data Center of the Chinese Acadamy of Sciences (CAS) [31, 32]. The watershed boundaries were delimitated based on the DEM data with the “automatic delineation utility” in BASINS. The Chaohu Lake Basin was divided into nine small watersheds; then we used GIS tools to calculate the area of each land use type within each subwatershed. Based on that we got the proportion of each land use area within each sub-watershed.

### 2.4. Spatial Patterns of Land Use

Landscape pattern change is mainly caused by the change in land cover and land use change [33]. The landscape ecologists and other researchers have developed numerous metrics to investigate the effects of the landscape pattern on the ecological processes [34]. In view of the multicollinearity among metrics and the erratic behaviors of some metrics across scales, we selected Shannon's diversity index (SHDI) as the indicator of landscape metric use in this study. SHDI indicates the patch diversity in a landscape based on the information theory, and it is calculated with the following form:

(1) $$\begin{array}{c} \text{SHDI}=-\sum_{i=1}^{m}\left(\text{pi}\;\;\ln\;\text{pi}\right), \end{array}$$

where pi is the proportion of the landscape occupied by land use type *i* and *m* is the number of land use type present in the landscape.

The SHDI is a sensitive indicator to analyze the diversity and heterogeneity of the same landscape in different times. The big value of SHDI means that the land use pattern is various and the degree of fragmentation is high. We calculated the percentages of the five land use types and the SHDI in these nine sub-watersheds and then analyzed the relationship between the SHDI and the indicators of water quality.

## 3. Results and Discussions

### 3.1. Descriptive Statistics of Measures

As showed in Table 2, the average CODmn concentration in 2000 and 2008 was 5.18 mg/L, with the concentration of NH3-N, TP, TN, and DO being 0.54 mg/L, 0.19 mg/L, 2.23 mg/L, and 8.22 mg/L, respectively. The standard deviation of indicators of water quality was generally very small.

The most important indicators of the water quality are the CODmn and TN, the average concentration of which reached 5.18 mg/L and 2.23 mg/L, respectively. The two indicators also vary greatly among the sub-watersheds, with their standard deviations being 1.09 and 1.11, respectively. There are main cultivated land and water areas in the Chaohu Lake Basin, accounting for 47% and 36% of the total area, respectively. Besides, the cultivated land and water area also vary most greatly among small watersheds, with their standard deviations reaching 0.12 and 0.16, respectively.

### 3.2. Water Quality Change in the Study Area

**Figure 2:**
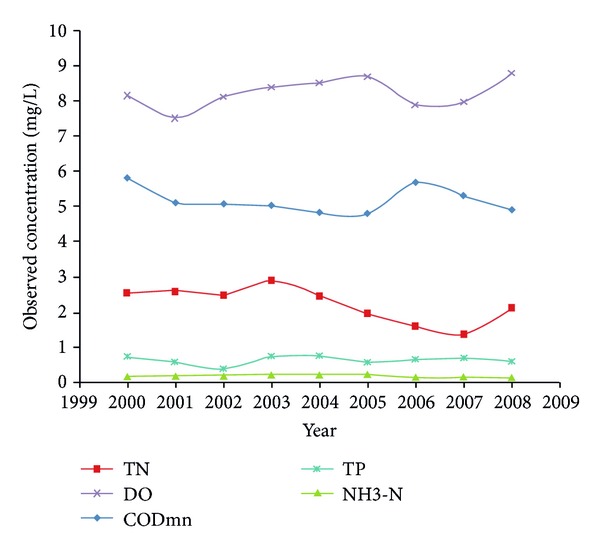
Water quality change from 2000 to 2008 in the study area.

According to the data from water quality monitoring points, water quality change in the study area was shown in Figure 2. In general, the water quality has improved from 2000 to 2008. Change trend of NH3-N and TP was small. And the change trend of DO was upward. The rest has changed a lot among this period; however, the beginning value was close to the finishing value.

### 3.3. Land Use Change

The overall state of land use in Chaohu Lake Basin was extracted based on the land use images (Table 3). The Chaohu Lake Basin is dominated by agriculture, and the cultivated land accounted for almost 60 percentage of the total area from 1995 to 2005. The total area of cultivated area has changed from 17153 km^2^ in 1995 to 16850 km^2^ in 2005, decreasing by about 1.7 percentage, while the area of built-up land increased by about 14 percentage. The urban expansion is the main driving factor of the decrease of cultivated land, and this kind of conversion would change the wetland soil which served as natural sinks and filtration system [35, 36]. The forest land accounted for approximately 20% of the total land area in the Chaohu Lake Basin, which was very stable in these years. The proportion of the grasslandand and water area in the Chaohu lake Basin was about 6.28% and less than 7%, respectively, both of which were very stable during 1995 and 2005. In this study, The unused land was not included in this study since it accounted for almost 0% of the total area.

### 3.4. Relationship between Land Use and Water Quality

The land use data and water quality monitoring data were analyzed by Stata. The model we used is an econometric model based on the research of four lakes watersheds in Hanyang district [18]:

(2) $$\begin{array}{c} \text{NPS}=\exp\left(\beta 1\times \text{land}1+\beta 2\times \text{land}2+\cdots \beta i\times \text{land}i\right), \end{array}$$

where NPS means the water quality variables in the study area, *α* is a constant, and *β* means the correlation between land use area (%) and water quality variables. When *βi* > 0, it means that land use type *i* has a positive effect on the indicators of water quality. If *βi* < 0, it means that land use type *i* has a negative effect on the indicators of water quality.

Since we got the data, the panel data has been used so as to comprehensively and completely reflect the relationship between the water quality and land use types in the Chaohu Lake Basin. Our panel data analysis was about 9 small watersheds in 2000–2008. Given the robustness and accuracy of the econometric analysis, we used both the fixed effect model and the random effect model so as to make a comparison, and finally the fixed effect model was selected according to the result of the Hausman test. Then we got the forms to describe the relationship between land use types and water quality according to the analysis results of Stata (Table 4):

(3) ln⁡⁡(TN)=−0.47C−4.11F−6.52G−0.3W+0.86B+1.2,ln⁡⁡(TP)=−0.42C−9.59F−7.87G−0.26W+1.54B−1.44,ln⁡⁡(CODmn)=−0.08C−5.47F−2.93G+0.29W+0.7B+1.69,ln⁡⁡(NH3−N)=0.31C−9.26F−8.83G+1.22W+2.6B−1.04,ln⁡⁡(DO)=0.06C+0.31F+0.54G+0.0029W−0.27B+2.08,

where *C* is cultivated land area (%), *F* is forest area (%), *G* is grassland area (%), *W* is water area (%), *B* is built-up area (%).

There was a positive relationship between the cultivated land area (%) and the concentration of NH3-N and DO. This is mainly due to the developed agriculture in the Chaohu Lake Basin and the emission of NH3-N from the exposure of soil surface resulting from the agricultural practices and the application of chemical fertilizers [37]. Besides, the concentrations of TP and TN are negatively related with the cultivated land area (%). On the one hand, the fertilizers used in the cultivated land will get into the runoff and flow into the river and ultimately pollute the river water. On the other hand, the vegetation in the surface soil of the cultivated land can absorb, retain the pollutants. As a result, the cultivated land plays a complicated role in influencing the water quality in the Chaohu Lake Basin.

The forest land and grassland both have significant positive influence on the water quality. The areas of the forest land and grassland were negatively related to TP, TN, NH3-N, and the CODmn and was positively related to DO. Many researches had shown similar results [15, 37].The significant negative relationship between the forest land and grassland area and TP, TN, CODmn, and NH3-N indicates that the the forest land and grassland played a key role in reducing the nitrogen pollutants and phosphorus pollutants and played a controlling role in regrating the water quality. The vegetation and soil in the forest land and grassland can effectively reduce the nutrient salts brought into the river by the surface runoff since they play an important role in reducing the surface runoff, conserving the water and soil, and absorbing the pollutants. Therefore, the increase of the forest land and grassland area will reduce the concentration of TP, TN, and oxygen-consuming substances, increase the concentration of dissolved oxygen, and consequently improve the water quality. 

The built-up area played a negative role in influencing the water quality on the whole. The built-up area was positively related to TP, TN, NH3-N, and CODmn and was negatively related to DO, indicating that the increase of the built-up area tends to degrade the water quality. Mouri et al. found similar relationship between concentration of TN and the area of built-up area [38], which is in agreement with that of Amiri and Nakane, who analyzed the relationship between TN, DO, NH3-N, and built-up area [37]. This could be the result of the increase of the nutrient concentration. The dense population density and economic activities both concentrated in the built-up area, which leads to very serious pollution. Besides, there is a lot of impermeable surface in the built-up area, which will contribute to the increase of surface runoff and may increase the concentration of nutrient salts in the river and consequently degrade the water quality within the watershed. There was a positive relationship between the built-up area (%) and TP and CODmn. The increase of built-up area was the result of transformation from the land with natural vegetation that could prevent the soil erosion. Since the vegetation can protect the soil from raindrops and tends to slow down the movement of runoff and allows the excessive surface water to infiltrate into soil, the conversion of the land with vegetation into built-up area will aggravate the soil erosion and consequently increase the amount of TP into the runoff.

### 3.5. Relationship between Spatial Patterns of Land Use and Water Quality

The relationship between SHDI and water quality was revealed in Table 5. SHDI had a negative relationship with NH3-N, TP, CODmn, and TN. According to the results of Stata analysis, we can know that the relationship between SHDI to CODmn, TP, TN, and NH3-N was very significant. The significant negative relationship mean that the landscape diversity in the Chaohu Lake Basin is closely related to the water quality. The higher the SHDI is, the greater the diversity landscape is and the slighter the deterioration of water quality is. As the landscape diversity increases, the landscape heterogeneity increases and consequently makes the patches of each landscape type more evenly distributed. 

## 4. Conclusion

Studying the relationship between the proportion of land use types and water quality in the Chaohu Lake Basin in this study indicated that built-up land was generally positively related to the indicators of water quality, and the forest land and grass land and water area were negatively related with the water quality variables, while the influence of the cultivated land on the water quality was very complex. Additionally, the built-up land, grassland, and forest land had significant influence on some indicators of water quality. The regression result of the landscape indicators and the indicators of water quality suggested that SHDI was negatively related to most of the water quality variables, indicating that the increase of landscape diversity can contribute to the improvement of water quality. 

According the result mentioned previously and the current conditions of the local water quality in the Chaohu Lake Basin, it is necessary to increase the area of forest land, grassland, and water area in the local land use planning. Since the forest land is more closely related to the local water quality, it is specially important to increase the area of forest land. Besides, the growth rate of the urban land should be slowed down under the condition of guaranteeing the minimum land area needed by the city development within the watershed. In addition, it is necessary to increase the landscape diversity because the greater the landscape diversity is, the more evenly patches of each kind are distributed and the more the water pollution will be alleviated.

The results of these studies can provide scientific reference for the local land use optimization and water pollution control and assist the formulation of policies for coordinating the water resource exploitation and protection. In addition, the previous researches have indicated that their landscape diversity has impacts on the water quality within the watershed, but it is still necessary to add some other ecological indicators and analyze their influence on the water quality. In particular, we should expand the method of analysing the relationship between land use and water quality but not the simple regression. Our study focuses on the effect of land use types and landscape patterns on water quality in the study area. However, there are many factors related to water quality, such as the climate, precipitation, and density of population. In the future work, we will refine the method and indicators to deeply reveal the reasons causing water quality change within a watershed.

## Figures and Tables

**Table 1:** Water quality of Chaohu Lake between 2000 and 2007.

River	2000	2001	2002	2003	2004	2005	2006	2007
Nanfei River	Bad V	Bad V	Bad V	Bad V	Bad V	V	V	Bad V
Shiwuli River	Bad V	Bad V	Bad V	Bad V	Bad V	Bad V	Bad V	Bad V
Pai River	Bad V	Bad V	Bad V	Bad V	Bad V	Bad V	Bad V	Bad V
Hangbu River	IV	II	III	III	IV	IV	III	IV
Baishitian River	IV	III	IV	III	IV	III	IV	IV
Zhao River	III	III	III	III	IV	IV	IV	IV
Tuogao River	III	III	IV	III	III	IV	III	III
Yuxi River	IV	III	III	IV	II	IV	III	IV
Shuangqiao River	Bad V	Bad V	Bad V	Bad V	Bad V	Bad V	Bad V	Bad V

**Table 2:** Descriptive statistics for the study area, including water quality and land use characteristics.

Categories	Variable	Obs	Mean	Min	Max	S.D.
Water quality	CODmn (mg/L)	81	5.18	2.7	7.8	1.09
	NH3–N (mg/L)	81	0.54	0.00	1.61	0.39
	TP (mg/L)	81	0.19	0.07	0.57	0.19
	TN (mg/L)	81	2.23	1.04	6.48	1.11
	DO (mg/L)	81	8.22	6.69	9.65	0.58

Land use	Cultivated land (%)	81	0.47	0.32	0.80	0.12
	Forest land (%)	81	0.02	0.00	0.06	0.02
	Grassland (%)	81	0.02	0.00	0.67	0.02
	Water area (%)	81	0.36	0.00	0.58	0.16
	Built-up area (%)	81	0.11	0.03	0.33	0.08

Landscape metrics	Shannon	81	2.83	1.76	3.85	0.50

S.D.: standard deviation.

**Table 3:** LUCC of the Chaohu Lake Basin between 1995 and 2005.

Year	Statistics variable	Cultivated land	Forest land	Grassland	Water area	Built-up land	Nonuse land
1995	Area (km^2^)	17153.11	5781.29	1817.64	2009.12	2165.91	1.32
	Proportion (%)	59.30%	19.98%	6.28%	6.95%	7.49%	0.00%
2000	Area (km^2^)	16960.22	5770.15	1817.34	2015.09	2364.22	1.32
	Proportion (%)	58.63%	19.95%	6.28%	6.97%	8.17%	0.00%
2005	Area (km^2^)	16850.80	5764.96	1816.65	2019.72	2474.93	1.32
	Proportion (%)	58.25%	19.93%	6.28%	6.98%	8.56%	0.00%

**Table 4:** The correlation coefficients between different land uses and water quality variables at the scale of the whole watershed.

Variables (%)	ln (TN)	ln (TP)	ln (CODmn)	ln (NH3–N)	ln (DO)
	coef.	coef.	coef.	coef.	coef.
Cultivated land	−0.46	−0.42	−0.08	0.31	0.06
Forest land	−4.11**	−9.59***	−5.47***	−9.26***	0.31
Grassland	−6.52***	−7.87***	−2.93***	−8.83***	0.54
Water area	−0.30	0.26	0.29*	1.22**	0.0029
Built-up area	0.86	1.54**	0.70**	2.60***	−0.27*
Constant	1.20***	−1.44***	1.69***	−1.04***	2.08***
*R*-squared	0.49	0.75	0.80	0.69	0.36

***P < 0.01, **P < 0.05, *P < 0.1.

**Table 5:** The correlation coefficients between landscape matrix and water quality variables at the scale of the whole watershed.

Variables (%)	ln (TN)	ln (TP)	ln (CODmn)	ln (NH3–N)	ln (DO)
	coef.	coef.	coef.	coef.	coef.
Shannon	−0.410***	−0.633***	−0.296***	−0.650***	0.0435***
Constant	1.866***	−0.00560	2.463***	1.232***	1.981***

***P < 0.01, **P < 0.05, *P < 0.1.
